# Modern craniopharyngioma management: three illustrative cases with distinct therapeutic strategies

**DOI:** 10.3171/2026.7.FOCVID26109

**Published:** 2026-10-01

**Authors:** A. Yohan Alexander, Crina M. Peterson, Matthew A. Tyler, Emiro Caicedo-Granados, Neal Godse, Kolin Rubel, Daniel J. Guillaume, Garrett Fitzpatrick, Andrew S. Venteicher

**Affiliations:** 1 Department of Neurosurgery and Center for Skull Base and Pituitary Surgery, University of Minnesota, Minneapolis;; 2 Department of Otolaryngology–Head and Neck Surgery, University of Minnesota, Minneapolis; and; 3 Department of Laboratory Medicine and Pathology, University of Minnesota, Minneapolis, Minnesota

**Keywords:** complex cranial, craniopharyngioma, endoscopic endonasal, microsurgery, molecular therapy, skull base

## Abstract

Craniopharyngioma treatment is increasingly multimodal, with options including surgery, radiation therapy, targeted molecular therapy (for papillary craniopharyngiomas), and a combination of these. As such, management of craniopharyngioma is complex, and strategies depend on patient-specific and tumor-specific factors. Nonetheless, literature regarding treatment selection is lacking. As such, this video details three distinct clinical scenarios of craniopharyngioma management. The authors specifically highlight the role of planned subtotal resection for endocrine preservation in adamantinomatous pediatric craniopharyngioma, gross-total resection for progressive adamantinomatous craniopharyngioma in an adult, and targeted molecular therapy for progressive symptomatic papillary craniopharyngioma.

The video can be found here: https://stream.cadmore.media/r10.3171/2026.7.FOCVID26109

## Transcript

This video demonstrates the modern management of craniopharyngioma with three illustrative cases.

### 0:24 Illustrative Cases.

We highlight the strategy of a planned gross-total resection in a progressive adult adamantinomatous craniopharyngioma; planned subtotal resection for endocrine preservation in a prepubertal adamantinomatous craniopharyngioma, and targeted molecular therapy for a progressive symptomatic papillary craniopharyngioma.

### 0:41 Illustrative Case 1.

The first case demonstrates the management of a progressive adamantinomatous craniopharyngioma. The patient was a 22-year-old morbidly obese female with progressively worsening vision. In these axial T1-weighted MRIs with contrast at an outside hospital, we can identify there is a heterogeneously enhancing, suprasellar mass with a large cystic component that extends into the middle fossa and anterior fossa. At the outside hospital, a right pterional approach was utilized to decompress the optic apparatus with an excellent visual outcome. The immediate postoperative MRI demonstrated decompression of the cyst but residual in the interpeduncular fossa. Five months later at our institution, repeat MRI demonstrated progressive growth of the lesion into the interpeduncular fossa, suprasellar cistern, and third ventricle. Her endocrine function was intact at this point. As the lesion was restricted to the midline and confined to the suprasellar cistern, interpeduncular fossa, and third ventricle, an endoscopic endonasal transtuberculum approach was utilized. Bilateral posterior clinoidectomies were performed for better maneuverability and visualization in the interpeduncular fossa.

### 1:44 Illustrative Case 1: Surgical Video.

Here we are looking at the posterior wall of the sphenoid sinus after bilateral posterior ethmoidectomies, sphenoidotomies, and a posterior septectomy. We start by thinning down the bone over the sella and flicking it away with a Kerrison. The midclivus is then partially drilled to afford access to the dorsum sellae. The bone over the right cavernous ICA and the left cavernous ICA are thinned down, and then the bone over the prechiasmatic sulcus is thinned down. Once the bone is thinned, we remove it again with a Kerrison to afford excellent dural exposure. We then open the interdural space with a feather knife and continue our inferior sellar periosteal incision posteriorly until the dorsum sellae is reached and the posterior clinoid is identified. After coagulation and division of the right inferior hypophyseal artery, we split the dorsum sellae and the right posterior clinoid is removed in a piecemeal fashion. On the left, we coagulate the inferior hypophyseal artery, divide it, and then dissect the posterior clinoid away from its ligamentous and dural attachments and remove it. We then open the sellar dura just under the superior intercavernous sinus. We then coagulate the superior intercavernous sinus and then divide the diaphragma sellae posteriorly. We can see the pituitary gland transposed to the left. We then open the cyst of the craniopharyngioma and internally debulk the lesion’s solid portions. Once debulked, we gently lift the tumor away from the basilar apex and identify the basilar bifurcation, the posterior communicating artery, the third nerve, and the right superior cerebellar artery. We then work on the medial aspect of the lesion, which interfaces with the infundibulum and the third ventricle. As we work posteriorly, we can identify the cerebral aqueduct at the posterior aspect of the lesion. Working laterally, we can identify the posterior communicating artery and its perforators at the lateral aspect of the lesion. Once these structures are identified, we carefully and sharply dissect the craniopharyngioma away from the walls of the hypothalamus and surrounding neurovascular structures. We then work on the superior aspect of the lesion at the inferior aspect of the chiasm; this is carefully and gently dissected away from the surrounding neurovascular structures, including the perforators coming from the posterior communicating artery. We continue to sharply dissect the lesion away from the walls of the hypothalamus and additional perforators. With traction and countertraction the lesion is carefully dissected away from the walls of the third ventricle, sharply disconnected of any attachments, and removed, affording gross-total resection. At the end of the resection, we can identify the anterior cerebral artery complex, the basilar bifurcation, a decompressed optic apparatus, and the third ventricle devoid of any tumor. The preserved pituitary stalk can be identified displaced to the patient’s left. Looking laterally, we can identify the right uncus between the posterior communicating artery and the third nerve. Reconstruction consisted of a DuraGen inlay, a fascia lata onlay, and a raised nasoseptal flap. This was bolstered with liquid dural sealant and absorbable nasal packing.

### 4:36 Illustrative Case 1: Postoperative Course, Imaging, and Follow-Up.

Postoperatively, the patient remained neurologically intact. On postoperative day 3, she did develop a CSF leak given her morbid obesity. This was managed by unclamping her previously placed lumbar drain for 4 days, which resulted in cessation of her CSF leak and no further treatment was required. She did require DDAVP for diabetes insipidus and hydrocortisone for cortisol insufficiency. Postoperative MRI revealed gross-total resection of the lesion. MRI at 5-year follow-up revealed no recurrent craniopharyngioma. Interestingly, at 1-year follow-up, she did not require any pituitary hormone replacement with no evidence of hypopituitarism. At 4-year follow-up, she was still endocrinologically intact with no need for DDAVP or hydrocortisone.

### 5:14 Illustrative Case 2.

The next case demonstrates planned subtotal resection in an adolescent with an adamantinomatous craniopharyngioma. The patient is an 8-year-old male who was found unconscious at home. He was endocrinologically intact at presentation. Axial CT at initial presentation revealed a hyperdense multicompartmental lesion. There is a rim of calcification around the lesion, and the lesion in the left sylvian fissure compresses the patient’s left central core. A right-sided EVD was placed with remarkable improvement in the patient’s neurological function. T1-weighted MRI with contrast at initial presentation revealed a large, relatively nonenhancing mass with a rim of peripheral enhancement. The lesion extends from the suprasellar space into the posterior fossa and left sylvian fissure. On the right axial image, one can appreciate the flow voids in the middle and posterolateral aspect of the lesion, suggesting encasement of the internal carotid and middle cerebral arteries, respectively. On axial T2-weighted MRI, one can appreciate that the lesion largely consists of an isointense cystic component with a solid hyperintense rim. A left-sided pterional approach with an extradural anterior clinoidectomy was utilized to access the lateral aspect of the lesion and avoid the infundibulum, which would have been centered in the endoscopic endonasal approach.

### 6:26 Illustrative Case 2: Surgical Video.

Here we are looking at the left sylvian fissure after the approach has been completed. We can see the temporal operculum to the left of the screen and the frontal operculum to the right of the screen. We begin by sharply incising the cyst, opening the incision with microscissors, and decompressing the cyst with suction. We then collapse the cyst wall away from the frontal and temporal opercula, and, as we dissect around the cyst wall, we can identify the M2 segment of the middle cerebral artery overlying the insula. The cyst wall is then removed in a piecemeal fashion. The lesion is then gently peeled away from the temporal pole, and where the lesion is tightly adherent to the surrounding parenchyma, it is carefully cauterized and divided. The lesion is then further debulked with microscissors. We then sharply dissect the lesion and peel it posteriorly to identify the tentorial incisura. As we continue to dissect, we identify the cyst in the posterior fossa and the supraclinoid internal carotid artery. Once these are identified, we continue to further debulk the solid portions of the lesion. We can identify the optic nerve at the anterior and inferior portions of the tumor. Gently elevating the tumor, we identify the ICA bifurcation into the anterior and middle cerebral arteries, the posterior communicating artery, and the optic nerve. With these structures well identified, we can continue to safely debulk the lesion. Once the sylvian portion has been debulked, we turn our attention to the interoptic space where we continue to open the capsule of the craniopharyngioma and internally debulk the lesion. In this region, we make sure to not dissect posteriorly in an effort to preserve the pituitary stalk and its function. We then turn our attention to the cyst in the posterior fossa and, working between the carotid artery and tentorial incisura, fenestrate the cyst and sharply open it with microscissors. At this point, an egress in CSF elucidates that the cyst is, in fact, filled with cerebrospinal fluid and not tumor extension. Following cyst decompression, we identify the posterior fossa structures of interest, including the pons, basilar artery, and the contralateral sixth nerve.

### 8:30 Illustrative Case 2: Postoperative Course, Imaging, and Follow-Up.

The patient remained neurologically intact postoperatively but had an aspiration pneumonia that was successfully treated with ceftriaxone. He had no have diabetes insipidus. He was discharged home on postoperative day 7. Immediate postoperative T1-weighted MRI with contrast revealed adequate decompression of the brain. In the middle sagittal image, we can see preservation of the pituitary stalk. At 2-month follow-up, the patient had an excellent endocrine outcome with normal TSH, prolactin, and IGF-1. We are closely monitoring his craniopharyngioma and endocrine function to determine the timing of any future treatment.

### 8:59 Illustrative Case 3.

The third case demonstrates the utilization of BRAF and MEK inhibitors for a progressive papillary craniopharyngioma. The patient was a 61-year-old female who had progressive subjective changes in vision and presented to her local ophthalmologist. She was endocrinologically intact. T1-weighted MRI with contrast at initial presentation revealed a heterogeneously enhancing sellar/suprasellar mass. An endoscopic endonasal transtuberculum approach was utilized as this lesion was confined to the suprasellar cistern and third ventricle.

### 9:28 Illustrative Case 3: Intraoperative Frozen Section.

Intraoperative results from frozen section suggested that this was a papillary craniopharyngioma given the squamous epithelium lining edematous fibrovascular cores. Thus, we stopped the resection after decompressing the optic apparatus. If the tumor progressed in the future, we would plan to treat with BRAF and MEK inhibitors.

### 9:44 Illustrative Case 3: Comparison of Papillary and Adamantinomatous Craniopharyngioma Histology.

Features of an adamantinomatous craniopharyngioma that the frozen section from our patient did not have include calcifications, stellate reticulum, keratin debris, and prominent basal nuclear palisading.

### 9:55 Illustrative Case 3: Postoperative Course, Imaging, and Follow-Up.

Postoperatively, the patient remained neurologically intact and had improvement in her visual acuity. She required transient use of DDAVP for diabetes insipidus that resolved. She was discharged home on postoperative day 5. Postoperative MRI revealed known subtotal resection of the lesion. Two months postoperatively, the patient had a sudden decrease in vision and repeat MRI revealed significant regrowth of the lesion and mass effect on the optic apparatus. In accordance with the previous plan, the patient was started on BRAF and MEK inhibitors immediately. We had a low threshold to pursue surgical decompression if vision did not improve. One-month following BRAF and MEK inhibitor therapy, the patient’s craniopharyngioma had decreased in size with improvement of mass effect on the optic apparatus and the patient’s vision had remarkably improved. In this graph demonstrating the patient’s visual function both on LogMAR and Ishihara color plates, we see the improvement of vision after initial surgery, the subsequent decrease in vision with tumor growth, and progressive and sustained improvement in visual function after BRAF and MEK inhibitor therapy. Six months after BRAF and MEK inhibitor therapy, the patient had sustained pyrexia without any evidence of infection. As such, medical therapy has been temporarily halted with no radiographic progression or clinical deterioration.

### 11:08 Illustrative Case 3: Role and Side Effects of BRAF/MEK Inhibitor.

Common side effects of BRAF/MEK inhibitor therapy include pyrexia, rash, fatigue, and diarrhea. Rarer risks include heart, kidney, or liver damage and cutaneous squamous cell carcinoma. BRAF and MEK inhibitor therapy may be used following maximal safe resection of a papillary craniopharyngioma either in the neoadjuvant or progressive setting, following biopsy of a papillary craniopharyngioma, or empirically in a radiographically suspected papillary craniopharyngioma in a poor surgical candidate. Radiographically, papillary craniopharyngioma can be suspected based on a predominantly suprasellar location, spherical shape, and homogeneous consistency and contrast enhancement with rare calcifications.^1^ Of course, papillary craniopharyngiomas are most common in the 5th and 6th decades of life.

### 11:57 Summary.

Modern management of craniopharyngioma is increasingly multimodal.^2–7^ For adults with adamantinomatous craniopharyngioma, we demonstrate the role of gross-total resection as a long-term tumor control strategy while preserving pituitary function in some cases. For adolescents with giant adamantinomatous craniopharyngiomas, we demonstrate the role of planned subtotal resection for endocrine preservation. Lastly, for progressive papillary craniopharyngiomas, we demonstrate that BRAF and MEK inhibitors can control tumor size and improve visual symptoms.

## Disclosures

M. Tyler reported consulting fees and honoraria from Sanofi/Regeneron outside the submitted work.

## Author Contributions

Primary surgeon: Venteicher, Tyler, Godse, Guillaume. Assistant surgeon: Peterson. Editing and drafting the video and abstract: Venteicher, Alexander, Peterson, Guillaume, Fitzpatrick. Critically revising the work: Venteicher, Caicedo-Granados, Godse, Rubel. Reviewed submitted version of the work: Venteicher, Peterson, Tyler, Caicedo-Granados, Godse, Rubel, Guillaume, Fitzpatrick. Approved the final version of the work on behalf of all authors: Venteicher. Supervision: Venteicher, Guillaume.

## Supplemental Information

### Patient Informed Consent

The necessary patient informed consent was obtained in this study.
